# Clinical impact of microbial cell-free DNA next-generation sequencing for invasive mold infection—a single-center retrospective observational study

**DOI:** 10.1177/20499361261437009

**Published:** 2026-04-03

**Authors:** Rebecca Berger, Nina Howard, Sarah Grant, Fernando Centeno, Todd Lasco, Mayar Al Mohajer

**Affiliations:** School of Medicine, Baylor College of Medicine, 1 Baylor Plaza, Houston, TX 77030, USA; School of Medicine, Baylor College of Medicine, Houston, TX, USA; School of Medicine, Baylor College of Medicine, Houston, TX, USA; Department of Medicine, Baylor College of Medicine, Houston, TX, USA; Department of Pathology, Baylor College of Medicine, Houston, TX, USA; Baylor St. Luke’s Medical Center, Houston, TX, USA; Department of Medicine, Baylor College of Medicine, Houston, TX, USA; Baylor St. Luke’s Medical Center, Houston, TX, USA

**Keywords:** *aspergillus*, immunosuppressed, microbial cell-free DNA, mucorales, non-invasive diagnostics

## Abstract

**Background:**

Diagnosing invasive mold infections (IMI) is challenging because they typically occur as opportunistic infections in immunocompromised patients who often present with non-specific symptoms. Furthermore, no single test can definitively diagnose IMI, and a proven diagnosis often requires invasive sampling. This sampling can be unsafe and difficult to perform, especially in the immunocompromised population most at risk for these infections.

**Objectives:**

The objective of this study is to assess the clinical utility of plasma microbial cell-free DNA next-generation sequencing (mcfDNA-NGS) for diagnosing invasive mold infections in the context of conventional diagnostic methods.

**Design:**

Retrospective observational study at a quaternary care center (2017–2025).

**Methods:**

The charts of 30 patients with mold-positive mcfDNA-NGS (Karius™ Spectrum; Redwood City, CA, USA) were reviewed, with IMI adjudicated per 2020 EORTC/MSGERC criteria. Provider documentation, medication orders, and patient outcomes were used to assess clinician interpretation of mcfDNA-NGS result accuracy (true positive vs false positive) and its impact on diagnostic and therapeutic decision-making. Turnaround time (TAT) and molecules per microliter (MPM) were summarized.

**Results:**

IMI final classifications were proven (6), probable (8), possible (5), and unclassified (11). Overall, 23/30 (77%) results were true positives. Among these 23 patients with true positive results for mold, 5 (22%) received a new clinical adjudication of IMI that had been entirely missed by conventional diagnostic testing. Furthermore, mcfDNA NGS provided species-level pathogen identification in 9 of the 23 (39%) true positive cases where conventional testing detected fungal elements or elevated biomarkers but could not identify the specific organism. Median TAT was 102 h; median MPM 657. MPM did not differ between true and false positives (*p* = 0.86). mcfDNA-NGS changed diagnostic classification in 7/30 (23%) and antimicrobial management in 16/30 (53%).

**Conclusion:**

mcfDNA-NGS provided noninvasive, actionable information, informing diagnosis and therapy. Future studies should define optimal stewardship and cost-effectiveness.

## Plain language summary

Clinical utility: Using DNA to find invasive mold infection

Some serious infections are caused by molds that are hard to treat. Usual tests can take a long time or may require risky procedures, such as lung or tissue biopsies. A new blood test called microbial cell-free DNA sequencing (mcfDNA-NGS) looks for pieces of fungus directly in the bloodstream, helping doctors identify infection faster. We looked at 30 patients with mold infections at our hospital. The blood test helped doctors make or change a diagnosis in 1 out of 5 patients and adjust antifungal treatment in nearly half. It also gives results within about four days. This study showed that mcfDNA-NGS blood test can give helpful information quickly and safely, especially for people whose immune systems are weak. More studies are needed to learn how best to use this test and how it affects patient recovery and costs.

## Background

Invasive molds, such as *Aspergillus*, *Mucor*, *Fusarium*, and *Scedosporium*, cause significant morbidity and mortality in immunocompromised patients.^1,2^ Early recognition is critical given the aggressive and disseminated nature of invasive mold infections (IMI), particularly in immunocompromised hosts. However, the diagnosis of IMI remains challenging, given their nonspecific clinical presentations. Moreover, no single diagnostic modality achieves both high sensitivity and specificity, necessitating the integration of clinical, radiologic, and mycologic data.^3,4^

The 2020 European Organization for Research and Treatment of Cancer/Mycoses Study Group Education and Research Consortium (EORTC/MSGERC) defines invasive mold infection as proven, probable, or possible based on host factors, clinical features, and mycologic evidence.^5^ Proven infection requires either culture of mold from a sterile site or histopathologic visualization of hyphae or yeast-like forms from a sterile site. Probable disease necessitates compatible host factors, clinical features, and mycologic data, whereas possible disease is defined by host and clinical findings alone.^5^

Mycologic testing remains central to diagnosis; however, it is hindered by several key limitations. Histopathology can confirm tissue invasion by the observation of microscopic fungal elements; however, obtaining tissue requires invasive biopsy that may be contraindicated in critically ill patients.^6,7^ Although culture is the cornerstone of diagnosis, it is slow (up to 4 weeks), technically demanding, and prone to contamination and overgrowth.^8,9^ Because molds are ubiquitous, a positive culture from non-sterile specimens may represent colonization rather than infection, particularly in non-immunocompromised hosts.^10^ These limitations underscore the ongoing need for diagnostic tools that are faster, less invasive, and more accurate.

Non-culture-based diagnostic assays have become increasingly prominent. For example, serologic tests such as galactomannan (GM) and 1,3-β-D-glucan (BDG) offer noninvasive adjuncts to diagnosis.^4,5^ Serological GM detection, specific to Aspergillus and occasionally *Fusarium*, has demonstrated a sensitivity of 60%–80% and a specificity of 80%–95%.^4^ BDG detects multiple fungal pathogens but lacks specificity and is best used as a supportive test. PCR-based assays further enhance specificity but require initial clinical suspicion and a standardized protocol.^4^

Plasma microbial cell-free DNA (mcfDNA) using next-generation sequencing (NGS) technology has recently emerged as a promising diagnostic modality for invasive mold infection.^11,12^ This technology enables broad hypothesis-free pathogen detection from a plasma sample, without prior knowledge of the causative organism. After automated extraction and library preparation, mcfDNA fragments are sequenced and compared against curated microbial databases to identify potential pathogens.^11^ Given its noninvasive nature and capacity for broad detection, mcfDNA-NGS represents a potentially transformative tool for diagnosing IMI in complex high-risk patients.

## Objectives

This study aimed to evaluate the clinical utility of mcfDNA-NGS for detecting IMI in a quaternary care setting. Specifically, the objective was to determine how mcfDNA-NGS results influenced diagnosis and the management of patients with suspected IMI. Secondary objectives included describing the spectrum of mold species detected, turnaround times (TATs), and concordance between mcfDNA-NGS findings and traditional diagnostic modalities.

## Design

**Figure 1.**
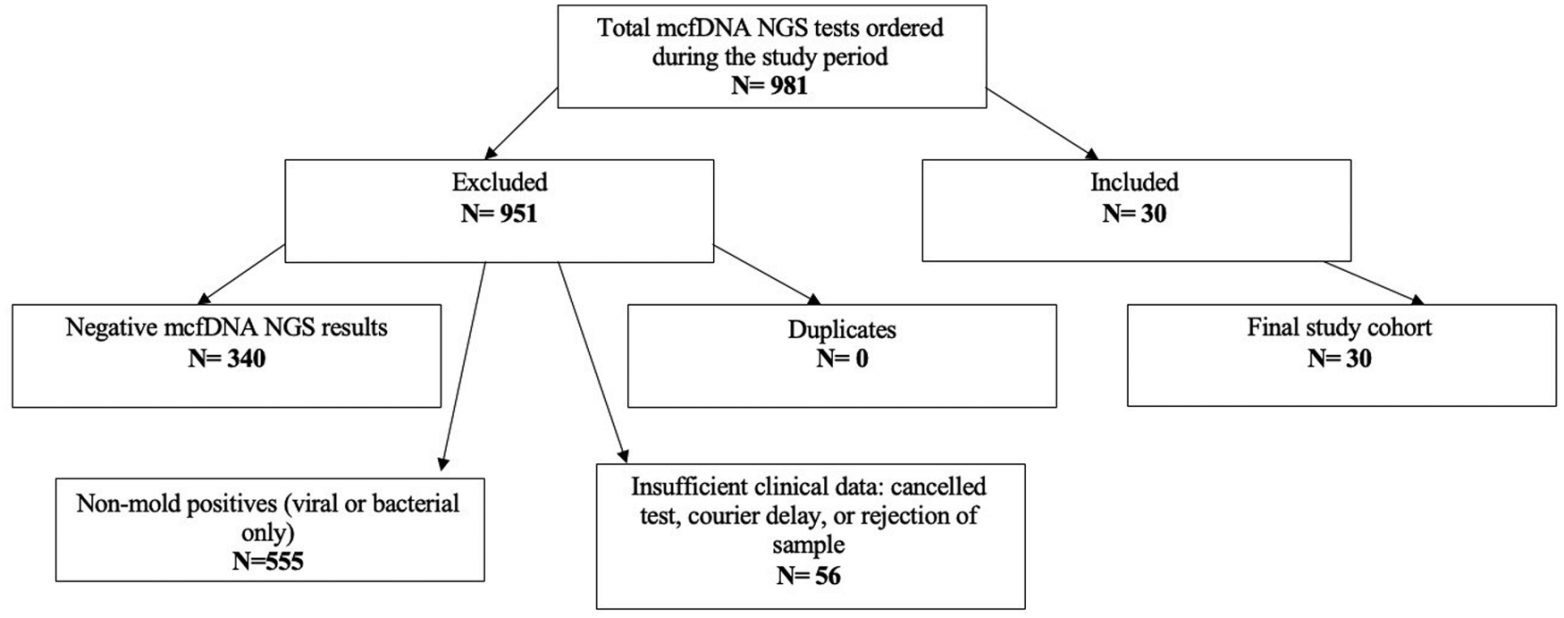
Flowchart showing inclusion and exclusion criteria for creating the cohort for this study. Of 981 total mcfDNA tests ordered during the study period, 30 met the inclusion criteria for final analysis. Major exclusion categories included negative results (*N* = 340) and non-mold positive results only (*N* = 555). mcfDNA, microbial cell-free DNA; *N*, number of samples; NGS, Next-Generation Sequencing.

This retrospective observational study was conducted at Baylor St. Luke’s Medical Center, a quaternary care center in Houston, Texas. Eligible patients in the cohort were those with positive mcfDNA-NGS results for mold between 2017 and 2025 (Figure 1). Any additional pathogens identified by mcfDNA-NGS were documented when present (Table 1). Given the absence of compiled data for initial suspected diagnoses at time mcfDNA NGS testing, we did not include negative results for patients who were suspected of IMI prior to testing. This study complied with the Strengthening the Reporting of Observational Studies in Epidemiology (STROBE) guidelines (Supplemental Table 1).^13^

**Table 1.** Microbiological testing.

Patient number	Mycologic evidence with conventional testing			mcfDNA NGS result: day from admission, TAT (hours), MPM (reference value)	First test to have a positive result	Clinical classification of mcfDNA positivity for mold
	Culture results (day from admission)	Microscopy results (day from admission)	BDG and GM results (day from admission)			
1	Epidural tissue fungal, culture (day 10): *Aspergillus fumigatus*	Epidural tissue, fungal smear (day 2): No fungi seen	BDG:NA and GM:NA	Day 8 (TAT: 41.3): *A. fumigatus*, 62 (<10)	mcfDNA NGS	True positive, given predisposing condition (uncontrolled diabetes), positive epidural fungal culture, and improvement on voriconazole
2	Right lung tissue, fungal culture (day 3): No growth	Right mainstem bronchus, fungal smear (day 3): True fungal hyphae with acute angle branching	BDG:NA and GM:NA	Day 14 (TAT: 99.27): *Rhizopus arrhizus*, <50 (NA)	Microscopy	True positive, given predisposing condition (uncontrolled diabetes), focal nodules on chest CT, hyphae visualization, and improving clinical status on appropriate antifungals
3	Ascites, fungal culture (day 1): No growth	NA	BDG:NA and GM:NA	Day 15 (TAT: 62.12): *Fusarium* species, 291 (NA)	mcfDNA NGS	True positive, given persistent fevers without obvious source and unexplained cytopenias with clinical improvement on targeted antifungals
4	NA	Nasopharynx/oropharynx tissue, fungal smear (day 9): Filamentous bacterial aggregates with surrounding inflammation	BDG (day 7): Inconclusive GM: NA	Day 9 (TAT: 135.78): *A. flavus*, 62 (NA)	Tie; mcfDNA NGS and microscopy	True positive given severe immunocompromised status, nodular opacities on CT chest, and clinical worsening without antifungals in setting of infectious presentation with broad antibiotic coverage
5	BAL, fungal culture (day 21): *A. fumigatus*	BAL, fungal smear (day 15): <1 budding yeast with pseudohyphae	BDG (day 23): >500, positive GM (day 17): 4.94, detected	Day 22 (TAT: 71.65): *A. fumigatus*, 20731 (NA)	Microscopy	True positive given immunocompromised status, nodular opacities on CT chest, and concordant conventional testing results
6	Sinus, fungal culture (day 9): *Candida albicans* Sinus, surgically obtained culture (day 13): Zygomycosis	Left orbital soft tissue and nasal septum, fungal smear (day 9): fungal hyphae Sinus, fungal smear (day 6): No fungi seen	BDG (day 18): <31, negative GM (day 8): <0.50, not detected	Day 8 (TAT: 96.97): *A. trapeziformis*, 5924 (NA) *A. variabilis*, 5643 (NA) *A. elegans*, 2337 (NA)	mcfDNA NGS and GM	True positive, given immunocompromised status and concordant conventional testing results
7	Sputum, culture (day 8): *Achromobacter* species Bronchus, culture (day 17): *Achromobacter* species BAL, fungal culture (day 12): *C. albicans* and *A. terreus*	Sputum, fungal smear (day 7): No fungi seen BAL, gram stain (day 13): normal respiratory flora BAL, fungal smear (day 12): No fungi seen	BDG (day 3): 50, negative GM (day 17): <0.50, not detected	Day 10 (TAT:90.07): *Mucor indicus*, 2932 (<10) *A. xylosoxidens*, *Klebsiella* spp.	mcfDNA NGS	False positive, given lack of findings on chest CT or via bronchoscopy, the positive mcfDNA NGS for *Mucor indicus* was determined to be due to colonization
8	BAL, bacterial culture (day 60): *A. calcoaceticus*, *Pseudomonas* species BAL (day 95), fungal culture: No growth	BAL (day 59), bacterial GMS: normal respiratory flora present BAL, fungal smear (day 95): No fungi seen	BDG (day 6, day 95): >500, positive GM (day 95): 8.95, detected	Day 97 (TAT: 65.43): *A. calidoustus*, not quantified due to low quantity	BDG	True positive, given immunocompromised status, positive BDG, GM, and worsening clinical status without appropriate antifungal coverage
9	Synovial fluid, left shoulder, fungal culture (day 1, 5): No growth	Synovial fluid, left shoulder, fungal smear (day 1, 5): No fungi seen	BDG:NA and GM:NA	Day 6 (TAT:126.1): *A. alternata*, 310 (<10)	McfDNA NGS	False positive, given lack of host factors, negative fungal smear and negative culture from the synovial fluid, the mcfDNA NGS result was termed to represent colonization
10	BAL, fungal culture (day 9): No growth	BAL, fungal smear (day 9): No fungi seen	BDG (day 1): >500, positive GM: NA	Day 4 (TAT:116.27): *A. fumigatus*, 61 (<10)	BDG	True positive, given immunocompromised status, history, and Aspergillus infection, and concordant positive BDG
11	Fungal culture (day 6): No growth	Fungal smear (day 6): No fungi seen	BDG:NA and GM:NA	Day 14 (TAT:129.55): *A. fumigatus*, 43 (<10) Bacteroides thetaiotaomicron (Bacteroides fragilis group), 485 (<10) *Pseudomonas aeruginosa*, 414 (<10) *P. distasonis* (Bacteroides distasonis), 238 (<10) *P. putida*, 148 (<50) *Bacteroides caccae* (Bacteroides fragilis group), 148 (<50) *Odoribacter splanchnicus*, 41 (<10) *Enterococcus faecium*, 47 (<10)	mcfDNA NGS	False positive, specifically for Aspergillus infection given comparatively lower MPM (to *Pseudomonas* spp. and Bacteroides spp.) Infectious was determined to be bacterial in nature, not fungal, and the mcfDNA NGS result was termed to represent colonization
12	BAL, fungal culture (day 20): *C. albicans*	BAL, fungal smear (day 11): No fungi seen	BDG (day 16): <31, negative GM (day 15): <0.50, not detected	Day 15 (TAT 78.03): *R. microsporus*, 657 (<10) *B. salyersiae* *Prevotella oris*	mcfDNA NGS	True positive, given predisposing condition (uncontrolled diabetes) and focal opacities on imaging
13	CSF, general culture (day 6): *A. fumigatus* CSF, fungal culture (day 3): No growth	CSF, fungal smear (day 4): hyphal elements seen	BDG (day 3): >500, positive GM: NA	Day 7 (TAT: 89.95): *A. fumigatus*, not quantified due to low quantity *Helicobacter pylori*	BDG	True positive, given immunocompromised status, focal CNS lesions on imaging, and concordance of CSF findings
14	Right-hand wound, culture (day 77): *Rhizopus* species and vancomycin-resistant enterococcus Paracenteses, fungal culture (day 1): No growth BAL, fungal culture (day 43): *C. albicans* BAL, fungal culture (day 65): No growth Right-hand wound, culture (day 68): vancomycin-resistant enterococcus faecium	Right-hand tissue debridement, surgical pathology exam (day 65): extensive necrosis with broad septate hyphae with angioinvasion Paracenteses, fungal smear (day 1): No growth BAL, fungal smear (day 37, day 65): No fungi seen Right-hand wound, gram stain (day 65): gram-positive cocci in clusters Right-hand wound, GMS (day 67): no organisms seen	BDG (day 40, day 57, day 68, day 70): 85, positive; 46, negative; 65, negative; 69, negative GM (day 64): <0.50, not detected	Day 69 (TAT: 73.2): *R. delemar*, 1768 (<10) *E. faecium* CMV, *Staphylococcus haemolyticus* (coagulase-negative staphylococcus) Herpes simplex virus type 1 (HSV-1)	BDG	True positive, given immunocompromised status due to decompensated cirrhosis and had concordant culture and microscopy results
15	Blood, fungal culture (day 36): No growth BAL, fungal culture (day 38): No growth	Blood, fungal smear (day 38): No fungi seen	BDG (day 39): >500, positive GM (day 47): 7.77, detected	Day 51 (TAT: 152.7): *A. fumigatus*, not quantified due to low quantity *E. faecium* CMV Torque teno virus *Legionella maceachernii*	BDG	True positive, given immunocompromised status, focal nodules on chest CT, and concordant BDG and GM results
16	Right leg eschar, fungal culture (day 41): *Fusarium* species Blood, fungal culture (day 7, day 32): No growth Blood, culture (day 40, day 42): *Fusarium* species*	Right leg eschar, fungal smear (day 36): hyphal elements seen Right leg eschar, punch biopsy (day 41): fungal elements with angioinvasion and tissue invasion and surrounding necrotic tissue Blood, gram stain (35): hyphal elements seen	BDG: NA GM (day 17, day 41); <0.50, not detected; 2.18, detected	Day 36 (TAT: 186.1): *Fusarium solani*, 9998 (<10) *Nectria haematococca* *E. faecium* *F. euwallaceae* Leuconostoc lactis *Janibacter indicus* EBV	Microscopy	True positive, given immunocompromised status, diffuse cutaneous eschars, and concordant culture and microscopy results
17	NA	NA	BDG:NA and GM:NA	Day 3 (TAT: 97.83): *Mucor circinelloides*, 166 (<10) CMV	mcfDNA NGS	False positive, termed to be colonization given absence of host factors/immunodeficiency or suspicious site of *Mucor*mycosis and lack of concordant microbiology findings
18	Right-hand abscess, fungal culture (day 9): *Scedosporium* species	Right-hand abscess, fungal smear (day 5): hyphal elements seen Tissue exam from right hand (day 5): necrosis and fungal hyphae seen	BDG (day 6): interfering substance present GM (day 6): <0.50, not detected	Day 3 (TAT: 85.13): *S. apiospermum*, 86 (<10) BK polyomavirus	mcfDNA NGS	True positive, given immunocompromised status, focal findings on imaging, and concordant culture and microscopy findings
19	Tissue from lung on autopsy, fungal culture (day 69): *A. terreus* Sputum, culture (day 43): *A. terreus* BAL, fungal culture (day 6, day 11): No growth	Tissue from lung on autopsy, fungal smear (day 46): hyphal elements seen Sputum, gram stain (day 42): normal respiratory flora seen BAL, fungal smear (day 6, day 11): No fungi seen	BDG (day 26): >500, positive GM (day 20): 3.87, detected	Day 20 (TAT: 169.93): *A. terreus*, 1013 (<10) *E. faecalis* EBV Herpes simplex virus type 1 (HSV-1)	mcfDNA NGS and GM	True positive, immunocompromised status, focal findings on imaging, and concordant findings on culture, microscopy, and BDG/GM
20	BAL, culture (day 9): *P. aeruginosa* BAL, fungal culture (day 2): No growth	BAL, fungal smear (day 2): No fungi seen	BDG (day 3): >500, positive GM (day 6): <0.50, not detected	Day 9 (TAT: 122.15): *A. fumigatus*, 35 (<10) *Klebsiella pneumoniae*	BDG	True positive, given immunocompromised status and cavitary air lesion on imaging
21	Sinus, culture (day 6): *A. fumigatus* Epidural tissue, culture (day 6): *A. fumigatus* Sinus, fungal culture (day 10): *A. fumigatus*	Epidural tissue, gram stain (day 3): No organism seen Sinus, fungal smear (day 3): No fungi seen	BDG:NA and GM:NA	Day 11 (TAT: 113.85): *A. fumigatus*, 62 (<10)	Culture	True positive, given predisposing condition (uncontrolled diabetes) and concordant culture results
22	BAL, fungal culture (day 4, day 9): No growth	BAL, fungal smear (day 4, day 9): No fungi isolated	BDG (day 10): 236, positive GM (day 9): <0.50, not detected	Day 15 (TAT: 117.92): *A. fumigatus*, not quantified due to low quantity EBV (Human herpesvirus 4)	BDG	True positive, given immunocompromised status, focal findings on CT, and positive BDG
23	BAL, fungal culture (day 7): *C. rugosa*, *C. glabrata*	BAL, fungal smear (day 4): No fungi seen	BDG: NA GM (day 11, day 17, day 21): 0.81, detected; 1.98, detected; 0.68, detected	Day 11 (TAT: 103.93): *A. terreus*, not quantified due to low quantity *Mycoplasma hominis* *B. cereus* *B. thuringiensis* *O. intermedium* *R. erythropolis* Torque teno virus Torque teno virus 1 Torque teno virus 27 Torque teno virus 28	mcfDNA NGS and GM	True positive, given immunocompromised status, focal findings on imaging, positive GM, and improvement on targeted antifungals
24	Sputum, culture (day 53): *Lichtheimia* species Blood, fungal culture (day 9, 11, 12): No growth Skin, fungal culture (day 11): No growth Body fluid from thoracentesis, fungal culture (day 42, day 44): No growth	Sputum, culture (day 41): normal respiratory flora seen Skin, fungal smear (day 11): No fungi seen Body fluid from thoracentesis, fungal smear (day 42, day 44): No fungi seen	BDG (day 17): 47, negative GM (day 12): 0.50, not detected	Day 16 (TAT: 140): *Lichtheimia ramosa*, not quantified *K. pneumoniae*	mcfDNA NGS	True positive, given immunocompromised status due to decompensated cirrhosis, focal findings and imaging, and concordant findings on culture
25	BAL, culture (day 80): *P. aeruginosa* Sputum, culture (day 2): *P. aeruginosa* Sputum, fungal culture (day 1): No growth Fluid from thoracentesis, fungal culture (day 4): No growth BAL, fungal culture (day 77): No growth	Sputum, fungal smear (day 1): No fungi seen Fluid from thoracentesis, fungal smear (day 4): No fungi seen BAL, fungal smear (day 77): No fungi seen	BDG (day 55, day 70): 174, positive; 91, positive GM: NA	Day 12 (TAT: 121.15): *A. alternata*, not quantified *E. cloacae* complex *K. pneumoniae* *P. aeruginosa*	McfDNA NGS	False positive, termed to be colonization given rare reports of this pathogen in immunocompromised hosts, and the patient was not immunocompromised, and the only concordant classic testing was the positive BDG, and this testing is non-specific
26	Wound from left leg, culture (day 15): *Fusarium* species Blood, fungal culture (day 6): No growth	Wound from left leg, gram stain (day 0): no organisms seen	BDG: NA GM (day 4): <0.50, not detected	Day 5 (TAT: 100.77): *A. nomius*, 398 (<10) *E. faecium* *Clostridium clostridioforme* *S. epidermidis* *Veillonella dispar* *Rothia mucilaginosa* *Corynebacterium amycolatum* *S. salivarius*	McfDNA NGS	True positive, given immunocompromised status, focal findings on imaging, and concordant culture results
27	BAL, fungal culture (day 14): *C. neoformans*	BAL, fungal smear (day 14): No fungi seen	BDG:NA and GM:NA	Day 3 (TAT: 39.8): *A. fumigatus*, <50 (NA) *Parabacteriodes merdae* *S. aureus* *E. faecium* EBV *Herpes simplex virus type 1* (HSV-1)	mcfDNA NGS	False positive, termed to be colonization given low Aspergillus MPM, steroid use, and improvement without antifungals
28	Drainage from ear, fungal culture (day 2, day 6): No growth Drainage from nose, culture (day 4): *P. aeruginosa, S. maltophilia*	Drainage from ear, fungal smear (day 2, day 6): No fungi seen Drainage from nose, gram stain (day 2): no organisms seen	BDG (day 9): 386, positive GM (day 10): <0.50, not detected	Day 9 (TAT: 65.23): *Aspergillus oryzae/flavus*, 62 (NA) *B. megaterium*	McfDNA NGS and BDG	True positive, given predisposing condition (uncontrolled diabetes), acute localized facial pain, and concordant BDG
29	BAL, fungal culture (day 10): No growth	BAL, fungal smear (day 10): No fungi seen	BDG (day 10): <31, negative GM (day 9): <0.50, not detected	Day 8 (TAT: 56.3): *L. prolificans* (formerly *S. prolificans*), 108 (NA)	mcfDNA NGS	True positive, given *L. prolificans* is an emerging opportunist infection and the patients immunocompromised status and focal findings on imaging
30	BAL, fungal culture (day 21): *Aspergillus* species	BAL, fungal smear (day 6): No fungi seen	BDG (day 11): 139, positive GM: NA	Day 13 (TAT: 123.7): *A. fumigatus*, 58 (NA)	BDG	False positive, patient was discharged before cultures grew *Aspergillus* spp. and mcfDNA NGS was positive for *Aspergillus* spp. However, there is no documentation that the patient was treated.

* Blood culture positivity indicates mold growth from blood and is distinct from mcfDNA NGS, which detects circulating cell-free fungal DNA without organism growth.

BAL, bronchoalveolar lavage; BDG, (1, 3)-β-D-Glucan assay; BK virus, Human polyomavirus 1; CMV, cytomegalovirus; CSF, cerebrospinal fluid; CT, computed tomography; EBV, Epstein–Barr Virus; GM, Galactomannan assay; GMS, Gomori Methenamine Silver stain; HSV-1, Herpes Simplex Virus Type 1; mcfDNA NGS, microbial cell-free DNA next-generation sequencing; MPM, molecules per microliter; NA, not applicable; spp., Species (plural).; TAT, turnaround time.

## Methods

For each patient, charts were reviewed from the admission during which mcfDNA-NGS testing occurred through the present date of review. Patient plasma specimens were sent to Karius (Redwood City, CA, USA) for analysis using the Karius™ Spectrum test, a plasma-based assay that identifies more than 1000 pathogens by detecting circulating microbial cell-free DNA. For each specimen, mcfDNA was isolated, sequenced, and compared with reference genomic databases to determine the presence of organisms.^12,14^

Results were reported as molecules per microliter (MPM), which refers to the quantity of DNA sequencing reads attributable to a specific organism detected in each microliter of plasma.^15^ MPM values are determined by comparing the number of sequencing reads attributed to a given organism with those of an internal control.^11^ Researchers only documented MPM values for mold pathogens identified via mcfDNA NGS (Table 1). Reference ranges varied by mold type, with many thresholds omitted due to the high risk of invasive disease at any detectable level (Table 1). Clinical TAT was calculated from the time of test order to result availability in the electronic medical record (Table 1).

## Data collection

Medical records were systematically reviewed for patient demographics, host factors, clinical presentation, imaging findings, mycologic evidence, antimicrobial management, and clinical outcomes (Tables 1–3). Besides mcfDNA NGS, additional diagnostics (e.g., imaging, microbiology, serology, and histopathology) were ordered at clinician discretion, and documented by reviewers. One investigator (R.B.) performed the initial chart review, and a second investigator (M.A.M.) independently verified accuracy. Discrepancies were resolved by consensus.

**Table 2.** Patient characteristics and clinical features.

Patient number	Age and sex	Initial signs and symptoms	Host factors according to the EORTC/MSGERC criteria	Clinical feature (key symptoms, exams, or radiography findings)	Mycologic evidence of mold according to the EORTC/MSGERC criteria	Classification of mold infection according to the European consensus criteria
1	Male, 69	Left frontal headache	No. However, the patient had poorly controlled diabetes (HbA1c: 9.1)	Yes 1. Acute localized pain 2. Sinus CT showing extension across bony barriers 3. Focal CNS lesion and meningeal enhancement on head CT	Yes.	Proven, given mold growth from sterile site
2	Male, 44	Fever and chronic cough	No. However, the patient had poorly controlled diabetes (HbA1c: 14.0)	Yes, chest CT showed complete right upper and middle lobe consolidation	Yes.	No classification, given absence of host factors according to the EORTC criteria
3	Female, 39	Fever of unknown source	No. However, the patient had decompensated cirrhosis	No	No.	No classification, given absence of host factors according to the EORTC criteria
4	Female, 53	Cough, dyspnea, rash, mucositis, fever	Yes, prolonged use of corticosteroids at a therapeutic dose of ⩾0.3 mg/kg for ⩾3 weeks in the past 60 days for interstitial lung disease, treatment with mycophenolate within 90 days	Yes, chest CT showed patchy nodular airspace opacities throughout both lungs	No.	Possible, given presence of host factors according and clinical features according to the EORTC, but absence of mycologic evidence
5	Female, 70	Dyspnea and hypoxia	Yes, history of bilateral lung transplant, prolonged use of corticosteroids at a therapeutic dose of ⩾0.3 mg/kg for ⩾3 weeks in the past 60 days, mycophenolate and cyclosporine within 90 days	Yes, chest CT showed bilateral nodular opacities	Yes.	Probable, given presence of host factors, clinical features, and mycologic evidence according to the EORTC criteria.
6	Male, 69	Chemosis and exophthalmos	Yes, chronic bronchitis requiring prolonged use of corticosteroids at a therapeutic dose of ⩾0.3 mg/kg for ⩾3 weeks in the past 60 days	Yes, maxillofacial MRI with infiltration of retroorbital space, and retro-maxillary fat	Yes.	Proven, given fungal hyphae visualization from sterile site
7	Male, 66	No symptoms	Yes, COPD requiring prolonged use of corticosteroids at a therapeutic dose of ⩾0.3 mg/kg for ⩾3 weeks in the past 60 days	No	Yes.	No classification, given absence of clinical factors according to the EORTC criteria
8	Female, 33	Dyspnea and hypoxia	Yes, status post bilateral lung transplant (for ILD secondary to SLE), treatment of mycophenolate, tacrolimus and hydroxychloroquine within 90 days	Yes, CT chest 11/6 showing nodules. No maxillofacial imaging	Yes.	Probable, given presence of host factors, clinical features, and mycologic evidence according to the EORTC criteria.
9	Male, 64	Left shoulder pain	No	No	No	No classification, given absence of host factors according to the EORTC criteria
10	Male, 31	Left leg weakness	Yes, s/p heart transplant and treatment with sirolimus and tacrolimus within 90 days	No. There were no classic imaging findings. However, MRI showed lumbar osteomyelitis	No.	No classification, given absence of host factors according to the EORTC criteria
11	Male, 69	Sternotomy pain and drainage	No	No	No	No classification, given absence of host factors according to the EORTC criteria
12	Male, 58	Dyspnea and hypoxia	Yes, prolonged use of corticosteroids at a therapeutic dose of ⩾0.3 mg/kg for ⩾3 weeks in the past 60 days	Yes, chest CT showing pulmonary nodules.	Yes.	Probable, given presence of host factors, clinical features, and mycologic evidence according to the EORTC criteria
13	Male, 44	Fatigue and generalized weakness	Yes, prolonged use of corticosteroids at a therapeutic dose of ⩾0.3 mg/kg for ⩾3 weeks in the past 60 days	Yes, CT head showing focal CNS lesions	Yes.	Proven, given fungal growth from sterile site
14	Male, 59	Right-hand wound	No. However, the patient had decompensated cirrhosis	No. There was classic signs or symptoms. However, the patient had tissue edema and fluid collection on hand CT	Yes.	Proven, given fungal hyphae visualization from sterile site
15	Female, 46	Fever and altered mental status	Yes, prolonged use of corticosteroids at a therapeutic dose of ⩾0.3 mg/kg for ⩾3 weeks in the past 60 days and treatment with mycophenolate mofetil, hydroxychloroquine, and eculizumab within the past 90 days (to treat SLE)	Yes, chest CT showing nodular opacities.	Yes.	Probable, given presence of host factors, clinical features, and mycologic evidence according to the EORTC criteria
16	Female, 46	Diffuse cutaneous eschars	Yes, Hodgkin's lymphoma and neutropenic for ten days due to Etoposide treatment	No. There was classic signs or symptoms. However, the patient had skin eschars	Yes.	Proven, given mold growth on sterile site and fungal hyphae visualization from sterile site
17	Female, 22	Arthralgia, headache, recurrent fevers, petechiae	No	No.	No	No classification, given absence of host factors according to the EORTC criteria
18	Male, 63	Vesicular rash with scattered pinpoint areas of eschars on the right arm	Yes, history of heart transplant and treatment with tacrolimus and mycophenolate mofetil within 90 days	Yes, chest CT from showing nodules in the right upper lobe. The patient also had skin eschars, but these are not considered a classic sign or symptom	Yes.	Probable, given the presence of host factors, clinical features, and mycologic evidence according to the EORTC criteria
19	Male, 40	Fever	Yes, neutropenia (unknown cause)	Yes, chest CT showing diffuse nodular opacities	Yes.	Probable, given presence of host factors, clinical features, and mycologic evidence according to the EORTC criteria
20	Female, 25	Anorexia, nausea, vomiting, right shoulder pain	Yes, history of bilateral lung transplant for CF, treatment with mycophenolate mofetil and tacrolimus within 90 days	Yes, chest CT showing cavity with air crescent sign	No.	Possible, given presence of host factors according and clinical features according to the EORTC, but absence of mycologic evidence
21	Male, 69	Left frontal headache	No. However, the patient had poorly controlled diabetes (HbA1c: 9.1)	Yes, head MRI with left frontal lobe cerebritis, intraparenchymal abscess, and subdural abscess	Yes.	Proven, given fungal growth from sterile site
22	Female, 62	Mucosal bleeding and fever	Yes, AML, neutropenia for over ten days, and treatment with Decitabine within 90 days	Yes, chest CT showing bilateral nodular opacities	No.	Possible, given presence of host factors according and clinical features according to the EORTC, but absence of mycologic evidence
23	Male, 68	Fatigue and anorexia	Yes, history of kidney transplant and treatment with tacrolimus within 90 days	Yes, chest CT showing scattered nodular opacities	Yes.	Probable, given presence of host factors, clinical features, and mycologic evidence according to the EORTC criteria
24	Male, 55	Abdominal and epigastric pain associated with nausea and vomiting	No, there were no classic host factors according to the European consensus criteria. However, the patient had decompensated cirrhosis	Yes, chest CT with bilateral lower lobe nodular opacities	Yes.	No classification, given absence of host factors according to the EORTC criteria
25	Male, 50	Dyspnea and hypoxia	No	Yes, chest CT showing bilateral nodular opacities	No	No classification, given absence of host factors according to the EORTC criteria
26	Female, 60	Fever and necrotic skin lesion on left anterior tibia	Yes, AML, neutropenia for over ten days, and treatment with Cytarabine and Daunorubicin within 90 days	Yes, chest CT showing consolidation in bilateral upper lobes	Yes	Probable, given presence of host factors according and clinical features according to the EORTC, and mold growth on culture of non-sterile site
27	Male, 71	Dyspnea and hypoxia	Yes, prolonged use of corticosteroids at a therapeutic dose of ⩾0.3 mg/kg for ⩾3 weeks in the past 60 days to treat rheumatoid arthritis	Yes, chest CT showing pulmonary cavitary	No	Possible, given presence of host factors according and clinical features according to the EORTC, but absence of mycologic evidence
28	Male, 82	Failure to thrive and nasal drainage in the setting of external ear pain and drainage	No. However, the patient had poorly controlled cites (HbA1c: 7.8)	Yes, acute, localized facial pain	No.	No classification, given absence of host factors according to the EORTC criteria
29	Male, 65	Fevers, fatigue, abdominal pain	Yes, AML, neutropenia for over ten days, and treatment with Decitabine and Venetoclax within 90 days	Yes, chest CT showing bilateral nodular opacities	No.	Possible, given presence of host factors according and clinical features according to the EORTC, but absence of mycologic evidence
30	Female, 53	Dyspnea and cough	No. However, the patient had severe emphysema and COPD	Yes, chest CT showing right-sided nodular opacities and cavitary lesions	Yes.	No classification, given absence of host factors according to the EORTC criteria

AML, acute myeloid leukemia; BLT, bilateral lung transplant; CF, cystic fibrosis; CNS, central nervous system; COPD, chronic obstructive pulmonary disease; CT, computed tomography; EORTC/MSGERC, European Organization for Research and Treatment of Cancer/Mycosis Study Group Education and Research Consortium; HbA1c, hemoglobin A1c; ILD, interstitial lung disease; MRI, magnetic resonance imaging; SLE, systemic lupus erythematosus; s/p, status post.

**Table 3.** Clinical impact of mcfDNA NGS testing.

Patient number	Change in diagnosis	Antimicrobial before mcfDNA NGS Result	Antimicrobial after mcfDNA NGS Result	Change in management from mcfDNA NGS mold result	Comparison: Classification of disease before and after mcfDNA NGS	Change in classification after mcfDNA NGS, compared to EORTC classification	Patient outcome (just leave as home even if had nursing)
1	No, proven invasive fungal disease made before mcfDNA NGS testing was ordered	Voriconazole, meropenem and vancomycin	Voriconazole	Yes, antibiotics were stopped	EORTC: Proven mcfDNA NGS: True positive	No	Improved and discharged home
2	No, team already suspected invasive fungal infection	Amphotericin B and cefepime	Posaconazole, amphotericin B and cefepime	Yes, posaconazole was started	EORTC: No classification mcfDNA NGS: True positive	Yes	Invasive fungal mass size decreased, but patient was discharged to inpatient hospice given multiple comorbidities
3	Yes, diagnosed with *Fusarium* infection without known source	Meropenem	Amphotericin B and voriconazole	Yes, amphotericin B and voriconazole were started, and meropenem was stopped	EORTC: No classification mcfDNA NGS: True positive	Yes	Discharged to home
4	No, test resulted posthumously	Penicillin for actinomyces	Micafungin and penicillin	Yes, Micafungin was started	EORTC: Possible mcfDNA NGS: True positive	No	Death from hemorrhagic shock due to spontaneous bleeding left lumbar artery which had previously undergone IR embolization
5	No, already suspected invasive fungal infection	Micafungin, voriconazole, meropenem, and vancomycin	Amphotericin B, daptomycin, meropenem, and vancomycin	Yes, amphotericin B and daptomycin were started and voriconazole and micafungin were stopped	EORTC: Probable mcfDNA NGS: True positive	No	Death due to septic shock and ischemic colitis
6	Yes, narrowed diagnosis to invasive fungal infection	Amphotericin B, metronidazole, cefepime, and vancomycin	Posaconazole and amphotericin B	Yes, posaconazole was started and metronidazole, cefepime, and vancomycin were stopped	EORTC: Proven mcfDNA NGS: True positive	No	Discharged to hospice facility after left eye exenteration and sinus surgery
7	No, mcfDNA NGS termed false positive for *Mucor* given lack of clinical correlation	Amphotericin B and piperacillin/tazobactam	Amphotericin B and piperacillin/tazobactam	No	EORTC: No classification mcfDNA NGS: False positive	No	Discharged to inpatient rehabilitation facility
8	Yes, diagnosed with Aspergillus ophthalmitis	Amphotericin B, itraconazole, ganciclovir, micafungin, voriconazole, levofloxacin, moxifloxacin, meropenem, sulfamethoxazole/trimethoprim, tobramycin, and vancomycin	Amphotericin B, itraconazole, ganciclovir, micafungin, voriconazole, levofloxacin, moxifloxacin, meropenem, sulfamethoxazole/trimethoprim, tobramycin, and vancomycin	No, although clinicians wanted to start IV amphotericin B (on inhaled) but patient died before initiating	EORTC: Probable mcfDNA NGS: True positive	No	Death to cardiac arrest shortly after mcfDNA resulted
9	No	Ceftriaxone, daptomycin, and vancomycin	Ceftriaxone, daptomycin, and vancomycin	No	EORTC: No classification mcfDNA NGS: False positive	No	Discharged to home
10	Yes, diagnosed with fungal lumbar osteomyelitis	Levofloxacin and vancomycin	Voriconazole, levofloxacin, and vancomycin	Yes, voriconazole was started	EORTC: No classification mcfDNA NGS: True positive	Yes	Transferred to another hospital per patient preference
11	Yes, made bacterial infection more likely	Cefepime, doxycycline, linezolid, meropenem	Daptomycin and meropenem	Yes, daptomycin was started and cefepime, doxycycline, and linezolid were stopped	EORTC: No classification mcfDNA NGS: False positive	No	Discharged to rehabilitation facility
12	No, already suspected invasive fungal infection	Meropenem, vancomycin, and voriconazole	Amphotericin B, daptomycin, and piperacillin/tazobactam	Yes, amphotericin B, daptomycin, and piperacillin/tazobactam were started while meropenem, vancomycin, and voriconazole were stopped	EORTC: Probable mcfDNA NGS: True positive	No	Death from acute hypoxic respiratory failure due to COVID-19
13	No, already confirmed invasive fungal infection prior to mcfDNA NGS result	Amphotericin B, ceftriaxone, rifaximin, and voriconazole	Amphotericin B, ceftriaxone, rifaximin, and voriconazole	No	EORTC: Proven mcfDNA NGS: True positive	No	Death due to multifocal fungal brain abscesses
14	No, already suspected invasive fungal infection	Amphotericin B, daptomycin, meropenem, posaconazole, and rifaximin	Amphotericin B, daptomycin, meropenem, posaconazole, and rifaximin	No	EORTC: Proven mcfDNA NGS: True positive	Yes	Discharged on hospice
15	No, test resulted posthumously	Ceftriaxone, ganciclovir, and voriconazole	None, test resulted posthumously	No, test resulted posthumously	EORTC: Probable mcfDNA NGS: True positive	No	Died from multiple cerebral hemorrhages due to antiphospholipid syndrome
16	No, already suspected invasive *Fusarium* infection	Amphotericin B, voriconazole, acyclovir, atovaquone, and meropenem	Amphotericin B, voriconazole, acyclovir, atovaquone, and meropenem	No	EORTC: Proven mcfDNA NGS: True positive	No	Death from cardiac arrest in the setting of invasive *Fusarium* infection, HLH, and Hodgkin's lymphoma
17	No, termed to be false positive for *Mucor* given lack of clinical correlation	None	None	No, termed to be false positive for *Mucor* given lack of clinical correlation	EORTC: No classification mcfDNA NGS: False positive	No	Discharged to home
18	No, already suspected invasive fungal infection	Amphotericin B and azithromycin	Voriconazole and azithromycin	Yes, started voriconazole and stopped amphotericin B	EORTC: Probable mcfDNA NGS: True positive	No	Discharged to skilled nursing rehabilitation center
19	No, already suspected invasive fungal infection	Amphotericin B and levofloxacin	Micafungin, voriconazole, and levofloxacin	Yes, started micafungin and voriconazole and stopped amphotericin B	EORTC: Probable mcfDNA NGS: True positive	No	Progressive fungal disease leading to massive pulmonary hemorrhage and death
20	No, already suspected invasive fungal infection	Sulfamethoxazole/trimethoprim, valganciclovir, posaconazole, piperacillin/tazobactam	Sulfamethoxazole/trimethoprim, Valganciclovir, posaconazole, piperacillin/tazobactam	No	EORTC: Possible mcfDNA NGS: True positive	No	Discharged to home
21	No, already suspected invasive fungal infection and the test resulted after discharge	Voriconazole	Voriconazole	No, patient was already on voriconazole treatment, and test resulted after discharge	EORTC: Proven mcfDNA NGS: True positive	No	Discharged to home
22	Yes, diagnosed with invasive fungal pneumonia	Amphotericin B, acyclovir, doxycycline, and meropenem	Voriconazole, meropenem, and acyclovir	Yes, started voriconazole while amphotericin B and doxycycline were stopped	EORTC: Possible mcfDNA NGS: True positive	No	Discharged to long-term acute care center
23	Yes, diagnosed with invasive fungal infection	Fluconazole, meropenem, sulfamethoxazole/trimethoprim, Vancomycin	Voriconazole, sulfamethoxazole/trimethoprim, and vancomycin	Yes, started voriconazole and stopped fluconazole	EORTC: Probable mcfDNA NGS: True positive	No	Discharged to home
24	No, already diagnosed with invasive fungal infection	Amphotericin B, azithromycin, doxycycline, meropenem, and vancomycin	Amphotericin B, doxycycline, meropenem, micafungin, and vancomycin	Yes, micafungin was started	EORTC: No classification mcfDNA NGS: True positive	Yes	Discharged to long-term acute care center
25	No, clinically termed to be false positive for *Mucor* given lack of clinical correlation	Cefepime and colistin	Cefepime and colistin	No, clinicians decided not to treat for fungal pathogens	EORTC: No classification mcfDNA NGS: False positive	No	Died from septic shock due to multidrug-resistant pseudomonal pneumonia
26	No, already diagnosed with invasive fungal infection	Piperacillin/tazobactam, posaconazole, valacyclovir, and vancomycin	Piperacillin/tazobactam, voriconazole, valacyclovir, and vancomycin	Yes, started voriconazole and stopped posaconazole and	EORTC: Probable mcfDNA NGS: True positive	No	Discharged to home
27	No, termed to be colonization from steroid use but clinically insignificant	Doxycycline, linezolid, and piperacillin/tazobactam	Doxycycline, linezolid, and piperacillin/tazobactam	No	EORTC: Possible mcfDNA NGS: False positive	Yes	Discharged to home
28	No, already suspected invasive fungal infection	Ciprofloxacin, meropenem, and minocycline	Ciprofloxacin, isavuconazonium, meropenem, and minocycline	Yes, started isavuconazonium sulfate	EORTC: No classification mcfDNA NGS: True positive	Yes	Discharged to home
29	No, already suspected invasive fungal infection	Cefepime, posaconazole, and valacyclovir	Levofloxacin, posaconazole, and valacyclovir	No, although stopped cefepime and started levofloxacin, but this was because of mcfDNA NGS result for bacterial pathogens	EORTC: Possible mcfDNA NGS: True positive	No	Discharged to home
30	No, clinical presentation was determined to be related to MAC and patient was discharged before mcfDNA NGS or cultures resulted	Azithromycin, ethambutol, and rifampin	Azithromycin, ethambutol, and rifampin	No, clinical presentation was determined to be related to MAC and patient was discharged before mcfDNA NGS or cultures resulted, and there was no documented treatment of invasive mold infection	EORTC: No classification mcfDNA NGS: False positive	No	Discharged to home

CT, computed tomography; EORTC/MSGERC, European Organization for Research and Treatment of Cancer/Mycosis Study Group Education and Research Consortium; MAC, Mycobacterium avium complex; mcfDNA NGS, microbial cell-free DNA next-generation sequencing; TMP/SMX, trimethoprim–sulfamethoxazole.

## Variable definitions

Mycologic evidence was assessed according to EORTC/MSGERC; definitions included: (1) culture of mold from a sterile site (e.g., blood, cerebrospinal fluid, tissue biopsy), (2) histopathologic visualization of fungal hyphae from sterile sites, (3) culture from non-sterile sites (e.g., bronchoalveolar lavage, sputum, wound specimens), (4) microscopy from non-sterile sites, and (5) positive GM antigen testing (Table 1).^5^ Although BDG results were documented, BDG positivity does not meet the EORTC/MSGERC definition as mycologic criteria (Table 1).^5^ Furthermore, mold-specific PCR assays were not available at the quaternary institution, and as a result, no corresponding PCR data were recorded.

Host factors were defined according to the EORTC/MSGERC criteria and included: (1) prolonged corticosteroid therapy (⩾0.3 mg/kg for ⩾3 weeks within 60 days prior to testing), (2) treatment with T-cell or B-cell immunosuppressants within 90 days, (3) solid organ transplantation, (4) hematologic malignancy, and (5) neutropenia (Table 2).^5^ Additional immunocompromising conditions not included in EORTC/MSGERC criteria—specifically uncontrolled diabetes and decompensated cirrhosis—were also documented given their clinical relevance to IMI risk (Table 2).^16–18^ The American Diabetes Association defines uncontrolled diabetes as a hemoglobin A1c (a measure of average blood glucose levels over the preceding 2–3 months) exceeding 7%.^18^ According to the American Gastroenterological Association, decompensated cirrhosis is characterized by the emergence of clinically evident complications, including ascites, hepatic encephalopathy, and/or variceal hemorrhage from the esophagus or stomach.^19^

Clinical features were defined per EORTC/MSGERC criteria and included pulmonary involvement (nodules, opacities, or consolidation on chest computed tomography (CT)), sinonasal or orbital involvement (extension across bony barriers on imaging), and central nervous system lesions (focal abnormalities on CT or magnetic resonance imaging (MRI); Table 2).^5^ Extrapulmonary manifestations such as soft tissue involvement, osteomyelitis, and cutaneous findings were also documented, though cutaneous findings are not recognized as clinical features under EORTC/MSGERC criteria (Table 2).

## Disease classification and outcome adjudication

Each case was classified according to the EORTC/MSGERC definitions of invasive mold infection (Table 2).^5^ The primary reviewer (R.B.) classified cases by comprehensive chart review, including patient demographics, previous diagnoses, presenting symptoms, working diagnoses at the time of mcfDNA-NGS testing as documented in clinical notes, vital signs, laboratory results, procedures, and official radiographic interpretations by institutional radiologists. Proven infection required either culture of mold from a sterile site or histopathologic visualization of hyphae in sterile tissue (Table 1).^5^ Probable infection required the presence of host factors as defined by EORTC/MSGERC, compatible clinical features, and mycologic evidence (Table 2).^5^ Possible infection was defined by the presence of host factors and clinical findings without mycologic confirmation (Tables 1 and 2).^5^ Cases that did not meet criteria for proven, probable, or possible infection were categorized as unclassified (Table 2).

Independent of EORTC/MSGERC classification, mcfDNA-NGS results were adjudicated as true positive or false positive based on treating clinician documentation in the electronic medical record (Tables 1–3). Evidence supporting true infection versus colonization was also corroborated by subsequent antimicrobial management decisions and documented clinical improvement or deterioration (Table 3).

Diagnostic impact was defined as any change in disease classification explicitly attributed to mcfDNA-NGS results in clinician documentation (Table 3). Therapeutic impact was defined as any antimicrobial modification (initiation, discontinuation, or change in antifungal agent) explicitly attributed to mcfDNA-NGS results in clinical notes (Table 3).

Patient outcomes were categorized as: discharged home, transferred to rehabilitation or long-term acute care facility, discharged to hospice, or death (Table 3). For deceased patients, documentation was reviewed to determine whether IMI was listed as the cause of death (Table 3).

## Statistical analysis

Quantitative variables were assessed for normality using visual inspection of histograms and the Shapiro-Wilk test. Given the small sample size (*n* = 30) and non-normal distribution of key variables, all continuous data were reported as median with interquartile range (IQR) or range, as appropriate (Table 4). TAT was measured in hours as a continuous variable. Due to the wide range of MPM values (spanning several orders of magnitude) and variation in reference ranges across mold species, MPM was not dichotomized or categorized for primary analyses (Table 4).

**Table 4.** Comparison of MPM values by clinical classification.

Parameter	True positive (*N* = 23)	False positive (*N* = 7)	Proven (*N* = 6)	Probable (*N* = 9)	Possible (*N* = 5)	No classification (*N* = 10)
Quantified samples	*N* = 18	*N* = 5	*N* = 6	*N* = 7	*N* = 4	*N* = 6
Not quantified	*N* = 5	*N* = 2	*N* = 0	*N* = 2	*N* = 1	*N* = 4
Median MPM	310	166	3,131	398	62	122
IQR	62–1768	58–310	657–6784	62–1768	58–166	50–310
Range	35–20731	43–2932	62–20,731	35–9998	58–291	<50–2932
Mean MPM	2461	722	6192	1994	144	779

IQR, interquartile range; MPM, molecules per million; *N*, number of samples.

Categorical variables were reported as frequencies and percentages. MPM values were compared between true positive and false positive cases to assess whether quantitative fungal DNA burden could distinguish infection from colonization. The Mann–Whitney *U* test was used, given the non-parametric distribution of MPM data. A two-tailed *p*-value < 0.05 was considered statistically significant. No adjustment for multiple comparisons was performed, considering the exploratory nature of this analysis.

Given the small sample size and descriptive nature of this study, formal multivariable adjustment for confounding was not performed. However, potential confounders were addressed through comprehensive clinical adjudication. The determination of true versus false positive mcfDNA-NGS results incorporated multiple factors, including host status, clinical presentation, imaging findings, conventional mycologic testing, clinical course, response to therapy, and treating clinician assessment (Tables 1–3). This integrative approach minimized the potential for confounding by indication (i.e., patients with more severe illness receiving more intensive diagnostic workup) and ensured that classifications reflected the totality of clinical evidence rather than any single factor.

Key potential confounders documented included: (1) non-EORTC/MSGERC immunocompromising conditions (uncontrolled diabetes, decompensated cirrhosis and (2) availability of tissue diagnosis (Tables 1–3). These factors were considered qualitatively when interpreting the results.

Diagnostic and therapeutic impact were descriptively examined by: (1) presence or absence of tissue confirmation, (2) EORTC/MSGERC classification category, and (3) specific mold species detected (Table 3). Formal statistical testing for interactions was not performed due to limited sample size and power.

Not every patient underwent all diagnostic modalities. These missing data reflect real-world clinical practice patterns where diagnostic testing was ordered at clinician discretion based on clinical suspicion, patient stability, and procedural risk. Missing diagnostic tests were not assumed to be negative; rather, the absence of testing was documented (Tables 1 and 2). This approach is appropriate for a descriptive cohort study and reflects the pragmatic clinical utility of mcfDNA-NGS in situations where conventional diagnostics are incomplete or unavailable.

All 30 patients were followed from their index hospitalization to final disposition (discharge home, transfer to rehabilitation/long-term acute care, discharge to hospice, or death; Table 3). No patients were lost to follow-up during the index hospitalization (Table 3).

## Results

There were 981 patients who underwent mcfDNA NGS testing at Baylor St. Luke’s Medical Center between 2017 and 2025 (Figure 1). Of these, 340 had negative mcfDNA NGS results, 555 were positive for non-mold pathogens on mcfDNA NGS testing, and 56 had no result due to cancellation, courier delay, or inadequate sample (Figure 1). There were no duplicate tests. Thirty patients tested positive for at least one mold pathogen on mcfDNA NGS testing and were included in this study (Figure 1).

The median age was 54.8 years (range: 22–82), and 11 (36.7%) were female (Table 2). Eighteen patients (60%) had at least one predisposing factor for IMI according to the 2020 EORTC/MSGERC criteria, with 11 (36.7%) exhibiting more than one risk factor (Table 2). Twelve (40.0%) received T-cell or B-cell immunosuppressants within 90 days before testing (Tables 2 and 5).^5^ Eight (26.6%) had received prolonged corticosteroid therapy, defined as a therapeutic dose of ⩾0.3 mg/kg for ⩾3 weeks within the 60 days prior to testing (Tables 2 and 5). Six (20%) were solid organ transplant recipients; four (13.3%) had hematologic malignancies, and five (16.7%) were neutropenic (Tables 2 and 5). Of the remaining 12 patients that did not have host factors according to the 2020 EORTC/MSGERC criteria, four (36%) had uncontrolled diabetes (patients 1, 2, 21, and 28) and three (25%) had decompensated cirrhosis (patients 3, 14, 24; Tables 1 and 2).

**Table 5.** Aggregate data summary.

Variable	*n* (%) or Median (IQR)
Age (years), median (range)	54.8 (22–82)
Sex, male	19 (63.3)
IMI disease classification	
Proven	6 (20.0)
Probable	8 (26.7)
Possible	5 (16.6)
Unclassified	11 (36.7)
Host factors^a^	
T- or B-cell immunosuppressant within 90 days	12 (40.0)
Prolonged corticosteroid therapy (⩾0.3 mg/kg ⩾ 3 weeks within 60 days)	8 (26.6)
Solid-organ transplant	6 (20.0)
Hematologic malignancy	4 (13.3)
Neutropenia	5 (16.7)
Uncontrolled diabetes or decompensated cirrhosis^b^	7 (23.3)
Common presenting symptoms	
Fever	8 (26.7)
Dyspnea	7 (23.3)
Localized pain	6 (20.0)
Cutaneous findings	5 (16.7)
Neurologic symptoms	3 (10.0)
Fatigue/anorexia/malaise	3 (10.0)
Cough	2 (6.7)
External ear drainage	1 (3.33)
Imaging findings consistent with IMI	
Pulmonary nodules/opacities/consolidation	16 (53.3)
Sinonasal/orbital involvement	3 (10.0)
CNS lesions	3 (10.0)
Bone or soft-tissue involvement (osteomyelitis, tibial lesion, shoulder, hand)	4 (13.3)
No abnormal imaging/imaging not performed	6 (20.0)
Mycologic evidence present	
Culture from sterile site	3 (10.0)
Fungal hyphae on histopathology from sterile site	3 (10.0)
Culture from a non-sterile site	10 (33.3)
Histopathology from non-sterile site	7 (23.3)
Positive BDG	12 (40.0)
Positive galactomannan antigen	6 (20.0)
No mycologic evidence	9 (30.0)
mcfDNA NGS species detected	
*Aspergillus* fumigatus	11 (36.7)
Other *Aspergillus* spp.	6 (20)
Mucorales spp.	7 (23.3)
*Fusarium* spp.	2 (6.7)
*Scedosporium*/*Lomentospora* spp.	2 (6.7)
*Alternaria* spp.	2 (6.7)

a Some patients had >1 host factor.

b Uncontrolled diabetes and decompensated cirrhosis are not included among EORTC/MSGERC host criteria but were present in several true IMI cases.

BDG, 1,3-β-D-glucan; CNS, central nervous system; EORTC/MSGERC, European Organization for Research and Treatment of Cancer/Mycosis Study Group Education and Research Consortium; IMI, invasive mold infection; mcfDNA, microbial cell-free DNA; NGS, next-generation sequencing.

Sixteen patients (53.3%) demonstrated pulmonary involvement based on chest CT findings (Table 5). Three patients (10%) had radiologic evidence of sinus and/or orbital involvement, and three (10%) had focal central nervous system (CNS) lesions (Table 5). Cutaneous involvement was observed in five patients (16.6%), including two with eschars (patients 16 and 18); however, cutaneous findings are not defined as clinical features per the 2020 EORTC/MSGERC criteria (Table 5).^5^ Three additional patients (10%) presented with soft tissue or bone involvement, including osteomyelitis and localized extremity lesions (Table 5).

Eighteen patients (60.0%) had mycologic evidence of infection, as defined by the 2020 EORTC/MSGERC criteria (Table 1).^5^ Mold was cultured from sterile sites in three cases, including epidural tissue specimens for patients 1 and 21 and cerebrospinal fluid for patient 13 (Tables 1 and 5). Patient 13 also had fungal elements visualized on microscopy of the CSF (Table 1). In three additional patients, molds were identified on histopathology of sterile specimens, including angioinvasive hyphae and fungal elements visualized in orbital tissue (Tables 1 and 5).

Mycological evidence from non-sterile specimens was identified in fifteen (50%) patients. Of these, twelve fulfilled EORTC/MSGERC mycologic criteria; the remaining three patients (patients 20, 25, and 28) had positive BDG as their sole mycologic finding, which does not meet the EORTC/MSGERC criteria (Table 1).^5^

Ten patients had mold growth from cultures of non-sterile sites: four from extremity wound cultures, three from sinus cultures, two from bronchoalveolar lavage, and one from lung tissue biopsy (Tables 1 and 5). Of these, patient 3 also had mold cultured from a different, sterile site (Tables 1 and 5). Fungal hyphae were visualized on microscopy of non-sterile sites in seven patients, two of which also had fungal hyphae identified in sterile site specimens (Tables 1 and 5). Twelve patients had positive BDG results, and six patients had positive galactomannan assays (Tables 1 and 5).

The median TAT for NGS testing was 102 h (interquartile range (IQR), 74–123; Table 1). mcfDNA-NGS identified five major mold classifications, the most common being the *Aspergillus* genera, detected in 17 patients, followed by molds within the Mucorales order (in seven patients; Table 1). The median MPM value for mold detections was 657 (IQR, 62–2293). The highest mean MPM level was observed among patients with proven infections according to EORTC/MSGERC criteria (Table 4).^5^ However, there was no statistically significant difference in the MPM values between true- and false-positive cases (*p* = 0.86; Table 4).

Based on the 2020 EORTC/MSGERC definitions, six patients met the criteria for proven IMI, eight for probable infection, and five for possible infection; the remaining 11 patients did not meet criteria for any classification of IMI (Tables 2 and 5).^5^ True positive mold infections were confirmed in 23 of 30 patients (77%) based on integration of host factors, clinical presentation, imaging findings, mycologic evidence, clinical course, and treating clinician assessment (Tables 1 and 3). The mcfDNA-NGS results in all proven and probable cases were clinically confirmed as true positive (Table 3). Of the five patients with possible invasive mold classifications, only one (patient 27) was determined to represent colonization rather than true infection (false positive; Table 3).

Among the 11 unclassified cases, five results were ultimately determined to be true positives (Table 3). Four of these lacked classic host factors as defined by the EORTC/MSGERC guidelines but were immunocompromised due to uncontrolled diabetes (patients 2 and 28) or decompensated cirrhosis (patients 3 and 24; Table 2). The final true positive patient, unclassified by EORTC/MSGERC criteria due to absent clinical findings, had MRI evidence of lumbar osteomyelitis (Tables 2 and 3).^5^ The remaining six patients that were not classified were considered false positives due to colonization (Tables 1 and 4).

Tissue confirmation was obtained in 13 patients (43.3%); five patients had positive cultures alone, one had fungal elements identified on microscopy alone, and the remaining seven patients had both culture and microscopy confirmation (Table 1). Of these 13 patients, 12 were classified as true positives and one as false positive (Tables 1 and 3). The remaining 17 patients had no tissue confirmation; of these, 10 were considered true positives based on non-invasive findings, including positive galactomannan antigen testing, concordant mcfDNA NGS results, and clinical course as interpreted by treating physicians (Tables 1 and 3).

Among all 30 patients, mcfDNA-NGS results led to a change in diagnostic classifications in seven cases (23.3%) and modification of antimicrobial management in 16 cases (53.3%; Table 3). Following hospitalization, 18 patients were discharged, including 14 who returned home and four who were transferred to rehabilitation or long-term acute care facilities (Table 3). Twelve patients died or were discharged to hospice, with IMI definitively listed as the cause of death in three cases (patients 13, 16, and 19; Table 3).

## Discussion

This is the first study to systematically evaluate the clinical impact of mcfDNA-NGS on the diagnosis and management of IMI among patients with varied causes of immunosuppression. Prior work has established the diagnostic potential of plasma mcfDNA-NGS for fungal disease, but few studies have examined how the test influences clinical decision-making. Our findings demonstrate that mcfDNA-NGS can meaningfully influence patient care, leading to diagnostic changes in 23.3% and therapeutic modification in 53.3% of cases (Table 4).

Other studies have shown that plasma mcfDNA-NGS can detect fungal DNA in patients with proven and probable IMI, with reported sensitivities ranging from 44% to 68% and specificities from 95% to 100%.^20–22^ Moreover, because tissue biopsy is often unsafe or infeasible in patients with hematological malignancy, transplantation, or critical illness, plasma mcfDNA-NGS offers a noninvasive alternative when conventional sampling cannot be performed.^23,24^ This advantage was corroborated by our findings, in which seventeen patients (57%) lacked tissue confirmation, of which 10 (58.8%) were clinically adjudicated as true positives based on concordant mcfDNA-NGS results and supporting radiologic or serologic evidence. A case-control study evaluating mcfDNA sequencing in high-risk immunocompromised patients reported higher sensitivity for proven versus probable invasive fungal disease (60.0% vs 37.1%)^25^ Although formal sensitivity calculations were not possible due to the absence of a control group, our results were concordant with prior findings: false positive results were more common in categories with lower diagnostic certainty according to EORTC/MSGERC criteria.

Quantitative dynamics of fungal mcfDNA have been evaluated in several longitudinal studies. Heldman et al.^23^ and Karam et al.^26^ demonstrated that MPM levels rose before clinical diagnosis and declined with antifungal therapy. In our series, the highest MPM levels occurred in patients with proven infections. However, there was no significant correlation between MPM values and clinical classification (true vs false positive), indicating that quantitative mcfDNA burden alone does not distinguish infection from colonization. These findings support prior observations that MPM is most informative when interpreted longitudinally rather than as an absolute cutoff.^23^

Our data also highlight persistent gaps between molecular diagnostics and classification frameworks. Of the 11 patients who were unclassified under the EORTC/MSGERC criteria, five were determined to have true positive IMI (Table 4). To receive a classification, patients must have specific host factors under the EORTC/MSGERC criteria.^5^ Four patients remained unclassified despite having significant immunocompromising conditions: two had uncontrolled diabetes, and two had decompensated cirrhosis. Both diagnoses are known to cause immunodeficiency leading to IMI, but were excluded from the criteria.^5,16–19,27–31^

One exhaustive case–control review of 925 patients with zygomycosis since 1885 found that 36% of patients with IMI had diabetes as their sole host risk factor, representing the most common underlying condition for IMI.^27^ This discovery corresponds with our findings, as one unclassified patient had uncontrolled diabetes and invasive mucormycosis. The other patient with pulmonary aspergillosis also lacked recognized EORTC/MSGERC host factors, yet this presentation has been documented in case series of patients with uncontrolled diabetes and no other risk factors.^28,29^ A systematic review and meta-analysis further demonstrated that ICU patients with diabetes mellitus were more likely to develop invasive aspergillosis than those without diabetes.^30^

Regarding cirrhosis and IMI, a study of 185 patients identified 19 cases of IMI after excluding all other types of immunodeficiency.^16^ The two patients in our cohort with cirrhosis who had true positive IMI but lacked EORTC/MSGERC-defined host factors had decompensated cirrhosis. This aligns with a meta-analysis showing that IMI was more common in patients with decompensated cirrhosis compared to those with compensated cirrhosis, whose IMI rates did not differ from the general population.^17^ Another report examining hematologic malignancy associated with pulmonary IMI emphasized that the rigid definitions may underestimate disease in the molecular era.^32^ Updating the consensus framework to incorporate mcfDNA-NGS, as well as decompensated cirrhosis and uncontrolled diabetes as host factors, could be essential to accurately capture the full spectrum of modern fungal disease.

Careful attention should be paid to the potential for false positivity when interpreting the mcfDNA-NGS results. Around a quarter (7/30) of the patients in this study had false positive results, which is consistent with other reports. Benamu et al.^33^ reported irrelevant mcfDNA in 29% of the patients with febrile neutropenia. The false positive rate of approximately 25% in our cohort underscores the need for judicious interpretation. Multiple mechanisms may contribute to false positivity, including transient mold DNAemia from mucosal barrier disruption, airway colonization releasing cell-free DNA without tissue invasion, residual DNA from previously treated infections, and potential low-level contamination despite rigorous laboratory protocols.^33^ To optimize clinical utility, mcfDNA-NGS results should be interpreted within a comprehensive diagnostic framework. Positive results, particularly low-level detections, require correlation with host factors (including non- EORTC/MSGERC immunocompromising conditions such as uncontrolled diabetes and decompensated cirrhosis), compatible clinical and radiologic findings, and conventional mycologic evidence when available. Serial MPM monitoring may help distinguish active infection from colonization or clearance, though our data suggest single MPM values alone do not reliably differentiate these states. Results obtained during or shortly after antifungal therapy should be interpreted cautiously, as they may represent treated disease rather than active infection. Antimicrobial stewardship programs should establish institutional guidance for mcfDNA-NGS utilization, including pretest probability assessment, multidisciplinary result interpretation involving infectious diseases expertise, integration with rather than replacement of conventional diagnostics, and quality improvement tracking to refine testing algorithms over time. Such stewardship efforts are essential to maximize diagnostic yield while minimizing overinterpretation and unnecessary antimicrobial exposure.

This study has several limitations. Only patients with positive mcfDNA NGS results were included because data on clinical suspicion for IMI at the time of test ordering were not systematically documented, precluding inclusion of all tests ordered for suspected IMI. Furthermore, since only patients with positive mcfDNA-NGS results were included, sensitivity and specificity could not be determined. Additionally, this study did not include a formal a priori sample size calculation. The sample size was determined by the availability of all mold-positive mcfDNA-NGS cases at our institution during the study period (*n* = 30), rather than by statistical power considerations. Given the exploratory nature of this investigation and the lack of established effect size estimates for mcfDNA-NGS diagnostic and therapeutic impact in IMI at the time of study design, a convenience sampling approach was utilized. The resulting sample size limits statistical power for subgroup analyses, precludes robust evaluation of test performance across different patient populations or mold species, and increases the risk of type II error when comparing quantitative parameters such as MPM values between groups.

PCR testing was not available at the study institution and therefore was not performed in any patient. This limitation is significant because PCR is included in the EORTC/MSGERC IMI criteria and, according to a systematic review, has demonstrated high sensitivity for detecting invasive mold infections, especially *Aspergillus* species.^34^

Additionally, cost-effectiveness was not evaluated, so the financial and operational impact of incorporating mcfDNA-NGS testing into routine practice remains uncertain. Despite these limitations, our clinician-verified documentation of how mcfDNA-NGS results influenced diagnostic and therapeutic decisions provides real-world evidence bridging analytic validation with bedside implementation.

The generalizability of this study’s findings is moderate and context-dependent. As a single-center, retrospective observational cohort conducted at a quaternary academic medical center, the results are most applicable to tertiary and quaternary care settings that manage complex, immunocompromised patients and have access to infectious diseases expertise, advanced imaging, and antifungal stewardship support. External validity is supported by the real-world nature of test ordering and interpretation, the heterogeneous patient population with varied immunosuppressive conditions and clinical presentations, and the inclusion of a broad range of invasive mold pathogens beyond Aspergillus alone. However, generalizability is limited by the small sample size, inclusion of only patients with mold-positive mcfDNA-NGS results, reliance on clinician adjudication rather than a uniform microbiologic gold standard, and institution-specific diagnostic workflows and TATs. Additionally, findings are specific to a single commercial mcfDNA-NGS platform and may not directly translate to other metagenomic assays. Consequently, while the results provide clinically relevant insights into the utility of mcfDNA-NGS in high-risk hospital settings, caution is warranted when extrapolating these findings to community hospitals, lower-risk populations, or screening contexts, underscoring the need for larger, multicenter prospective studies to confirm broader applicability.

In conclusion, plasma mcfDNA-NGS is an important advance in non-invasive mold diagnostics. Our findings show that mcfDNA-NGS results can guide diagnosis and management, highlighting the need for prospective studies to define its optimal clinical and economic role.

## Supplemental Material

sj-docx-1-tai-10.1177_20499361261437009 – Supplemental material for Clinical impact of microbial cell-free DNA next-generation sequencing for invasive mold infection—a single-center retrospective observational study Supplemental material, sj-docx-1-tai-10.1177_20499361261437009 for Clinical impact of microbial cell-free DNA next-generation sequencing for invasive mold infection—a single-center retrospective observational study by Rebecca Berger, Nina Howard, Sarah Grant, Fernando Centeno, Todd Lasco and Mayar Al Mohajer in Therapeutic Advances in Infectious Disease

## Declarations

**Ethics approval and consent to participate:** The study was approved by the Baylor College of Medicine Institutional Review Board (approval number H-45143). Informed consent requirement was waived, given the retrospective nature of the study.

**Consent for publication:** Waived by the Baylor College of Medicine IRB. The study used existing records from a multi-year cohort; re-contacting all patients was not practicable because many were deceased, lost to follow-up, or no longer receiving care at our center. Only de-identified data were analyzed.

**Availability of data and materials:** The data and materials accessed for this study contain identifiable patient information and are, therefore, only available to providers and for research purposes.
